# Evolution of an adaptive behavior and its sensory receptors promotes eye regression in blind cavefish: response to Borowsky (2013)

**DOI:** 10.1186/1741-7007-11-82

**Published:** 2013-07-11

**Authors:** Masato Yoshizawa, Kelly E O’Quin, William R Jeffery

**Affiliations:** 1 Department of Biology, University of Maryland, College Park, MD, 20742, USA

**Keywords:** Animal behavior, Regressive evolution, Constructive evolution, Neuromast, Tradeoff, Pleiotropy, Quantitative trait locus, Eye, QTL cluster, Adaptation

## Abstract

Vibration attraction behavior (VAB) is the swimming of fish toward an oscillating object, a behavior that is likely adaptive because it increases foraging efficiency in darkness. VAB is seen in a small proportion of *Astyanax* surface-dwelling populations (surface fish) but is pronounced in cave-dwelling populations (cavefish). In a recent study, we identified two quantitative trait loci for VAB on *Astyanax* linkage groups 2 and 17. We also demonstrated that a small population of superficial neuromast sensors located within the eye orbit (EO SN) facilitate VAB, and two quantitative trait loci (QTL) were identified for EO SN that were congruent with those for VAB. Finally, we showed that both VAB and EO SN are negatively correlated with eye size, and that two (of several) QTL for eye size overlap VAB and EO SN QTLs. From these results, we concluded that the adaptive evolution of VAB and EO SN has contributed to the indirect loss of eyes in cavefish, either as a result of pleiotropy or tight physical linkage of the mutations underlying these traits. In a subsequent commentary, Borowsky argues that there is poor experimental support for our conclusions. Specifically, Borowsky states that: (1) linkage groups (LGs) 2 and 17 harbor QTL for many traits and, therefore, no evidence exists for an exclusive interaction among the overlapping VAB, EO SN and eye size QTL; (2) some of the QTL we identified are too broad (>20 cM) to support the hypothesis of correlated evolution due to pleiotropy or hitchhiking; and (3) VAB is unnecessary to explain the indirect evolution of eye-loss since the negative polarity of numerous eye QTL is consistent with direct selection against eyes. Borowsky further argues that (4) it is difficult to envision an evolutionary scenario whereby VAB and EO SN drive eye loss, since the eyes must first be reduced in order to increase the number of EO SN and, therefore, VAB. In this response, we explain why the evidence of one trait influencing eye reduction is stronger for VAB than other traits, and provide further support for a scenario whereby elaboration of VAB in surface fish may precede complete eye-loss.

## 

Borowsky’s first two points involve the interpretation of specific experimental results, specifically the evidence for an relationship among vibration attraction behavior (VAB), neuromast sensors located within the eye orbit (EO SN) and eye size based on the overlap of quantitative trait loci (QTL) for these traits. Borowsky highlights several additional QTL on linkage groups (LGs) 2 and 17 that could also interact with eye size, including those for traits that are putatively adaptive (condition factor, maxillary teeth), maladaptive (maxillary teeth, weight loss, depth of caudal peduncle), and neutral (melanophore number, suborbital bone width, thoracic rib number). “Thus,” Borowsky writes, “there are numerous potential interactions in this region; none of which is individually strongly supported on the sole basis of the proximity of QTL.”

Contrary to Borowsky’s contention, we did not argue for the existence of an ‘exclusive*’* relationship among VAB, EO SN and eye size, only the existence of a ‘direct*’* relationship between VAB and EO SN and (possibly) an ‘indirect*’* one among these and eye size. We based these conclusions on several lines of evidence. First, we concluded that VAB and EO SN are directly related based on experimental evidence that EO SN ablation reduces VAB [1]. Second, we concluded that both may be indirectly related to eye size given (1) the position of the EO SN within the eye orbit, (2) the correlation of both VAB and EO SN with eye size among the members of our genetic cross (*r* = −0.26 and −0.44, respectively, both *P* <0.001), and (3) the significant clustering of all four QTL for VAB and EO SN with two of the five QTL for eye size in just two regions of the *Astyanax* genome, a pattern that is unexpected by chance assuming a Poisson distribution of QTL locations (*χ*^*2*^ = 98.2, *df* = 3, *P* = 3.8 × 10^-21^). Although we feel that these results offer strong evidence for our conclusions, their strength and significance may be best understood in comparison to the other QTL that Borowsky has highlighted.

First, we concede that other traits within this region are also genetically correlated with eye size. Several putatively adaptive traits exhibit strong genetic correlations with eye size (condition factor, weight loss and chemical sensing ability; *r* = −0.24, 0.17, and −0.32, respectively; all *P* <0.01), although many putatively neutral and maladaptive traits do not (suborbital bone width, depth of caudal peduncle, melanophore number and maxillary teeth) [2]. But in either case, these observations constitute neither evidence for nor against the existence of a direct relationship between VAB and EO SN or an indirect one among VAB, EO SN and eye size.

Second, our argument did not rely solely on the existence of overlapping QTL, but also on QTL clustering in a manner that was unlikely to be observed by chance. Importantly, when the overlap among Borowsky’s other putatively adaptive QTL is measured in the same manner, the observed overlap of condition factor, weight loss and maxillary teeth with the eye size QTL is not significantly different than expected by chance (*χ*^*2*^ = 2.0, *df* = 2, *P* = 0.359). The clustering of these additional traits does reach statistical significance if considered in terms of the multi-trait model used by Protas *et al*. [3] (*χ*^*2*^ = 13.1, *df* = 4, *P* = 0.0001); however, this model is different from the QTL strategy that we implemented and it implicitly assumes that traits are correlated as a result of pleiotropy or tight linkage [4] - the same conclusion we draw in our study. We acknowledge that the large distance between peaks for the eye and VAB QTL on LG2 may not provide the most convincing evidence for pleiotropy or hitchhiking under the assumption that the responsible mutations reside under the peak of each QTL; however, we only assume that they fall somewhere within the QTL’s 95% confidence limits.

Finally, of all the putatively adaptive traits that Borowsky implies could just as easily explain the correlated evolution of traits on LGs 2 and 17, there is only genetic or experimental evidence of adaptation for two: eye size and VAB [1,2]. We estimated that VAB provides a 47% increase in foraging efficiency under laboratory conditions [5]. Assuming that this advantage provides an estimate of the selection coefficient for VAB [6], so long as the selection coefficient of VAB and/or eye size remains substantially larger than the selection coefficients of nearby alleles that determine putatively neutral or even maladaptive traits, then these linked QTL can still be carried to fixation as a result of genetic hitchhiking [7,8].

Borowsky’s final two points concern the assumption that indirect selection is necessary to explain the evolution of eye loss among cavefish. Given the numerous tradeoffs between eyes and non-visual sensory systems that have been documented among cave organisms [9-11], we made this assumption explicit at the outset of our study. But, based on the consistent polarity of numerous eye QTL, Borowsky argues that no such assumption is necessary since the most parsimonious explanation for this observation is direct selection against eyes. Borowsky further argues that eye size must first be reduced in order to promote EO SN expansion and, therefore, VAB. Whether or not direct or indirect selection (or both) is ultimately responsible for cavefish eye-loss is beyond the scope of this reply, but we can address the validity of this assumption and our proposed evolutionary scenario.

We agree that the consistent polarity of cavefish eye QTL constitutes strong genetic evidence for selection against eyes. And although direct selection against eyes may be the simplest explanation for this observation, it is not necessarily the only one. In his paper describing the sign test used to infer the role of selection based on the polarity of QTL, Orr noted that, “rejection of the null hypothesis does not, strictly speaking, allow us to conclude that the analyzed character was the direct target of selection. One can never completely exclude the possibility that the measured character changed as a correlated response to selection (although this seems less plausible for the larger, and sometimes dramatic, character differences often considered in QTL analyses)” [12]. Thus, indirect selection against eyes is not ruled out on the basis of QTL polarity.

As for the plausibility that VAB promotes eye loss before eyes have been completely reduced, we wish to highlight three important points. First, we note that some lab-reared and fully-eyed *Astyanax* surface fish exhibit a weak form of VAB [5,13], suggesting that this trait may be present at low frequencies in natural populations. Second, competition assays between these VAB-positive and VAB-negative surface fish confirm that VAB-positive surface fish out-compete VAB-negative ones and that this advantage disappears under lighted conditions, supporting the foraging advantage of VAB in darkness even among surface fish [5]; furthermore, VAB was abolished when VAB-positive surface fish were treated with lateral line inhibitors, suggesting that surface fish VAB also function through the lateral line system [5,13]. Third, we also found that the number of EO SN is negatively correlated with eye size among eyed F_2_ and F_3_ progeny. These three observations suggest that VAB and EO SN number can increase foraging efficiency even among *Astyanax* eyed populations [1]. Since surface fish with VAB lack EO SN but nonetheless respond weakly to a broad range of vibrations between 5 and 35 Hz [13], the enhanced EO SN and 35 Hz VAB tuning found among cavefish might have evolved following some initial reduction in eye size among VAB-positive surface fish populations that invaded caves [13].

We acknowledge that other factors can and do promote eye loss in *Astyanax*. In Yoshizawa *et al*. [1], we demonstrate that the proposed VAB-EO SN pathway is independent of the pleiotropic SHH-pathway that has already been shown to influence cavefish eye degeneration [14]. Here, we also note that there are cavefish populations that exhibit eye reduction without exhibiting VAB, especially those found in the Molino and Tinaja caves [5]. But these observations do not contradict the conclusions of our study. It is well established that eye-loss has evolved more than once among different *Astyanax* cavefish populations [15-20], and several lines of evidence suggest that different mutations are responsible for eye-loss among these groups [17,21]. We did not propose that all cases of eye-loss in *Astyanax* are the result of VAB or even that VAB is directly responsible for eye reduction. Rather, we proposed that VAB may have promoted eye-loss due to direct selection on nearby loci for VAB. If the nearby eye QTL are not under direct selection (as Borowsky suggests may be the case for the QTL on LG 17), then eye-loss could have evolved indirectly via pleiotropy or hitchhiking, perhaps following some initial reduction in eye size due to direct selection at several other eye QTL. Alternatively, if both VAB and the nearby eye QTL are under direct selection, then eye reduction and VAB could have evolved together due to the combined fitness of their alleles. Unfortunately, our current results cannot distinguish between these two evolutionary scenarios. In the end, we agree with Borowsky that “the hypothesis may or may not be true, but we will not know until the genes are identified and characterized.” And, thanks to the active development of new genomic resources for *Astyanax*, the answer may come sooner rather than later.

## Abbreviations

EO: Eye orbit; LG: Linkage group; QTL: Quantitative trait locus; SN: Superficial neuromast; SO-3: Suborbital bone 3; VAB: Vibration attraction behavior.

## Competing interests

The authors declare that they have no competing interests.

## References

[B1] Yoshizawa M Yamamoto Y O’Quin KE Jeffery WR Evolution of an adaptive behavior and its sensory receptors promotes eye regression in blind cavefish BMC Biol 2012 10 108 10.1186/1741-7007-10-108 23270452 PMC3565949

[B2] Protas M Conrad M Gross JB Tabin C Borowsky R Regressive evolution in the Mexican cave tetra, *Astyanax mexicanus* Curr Biol 2007 17 452 454 10.1016/j.cub.2007.01.051 17306543 PMC2570642

[B3] Protas M Tabansky I Conrad M Gross JB Vidal O Tabin CJ Borowsky R Multi-trait evolution in a cave fish, *Astyanax mexicanus* Evol Dev 2008 10 196 209 10.1111/j.1525-142X.2008.00227.x 18315813

[B4] Korol AB Ronin YI Itskovich AM Peng J Nevo E Enhanced efficiency of quantitative trait loci mapping analysis based on multivariate complexes of quantitative traits Genetics 2001 157 1789 1803 11290731 10.1093/genetics/157.4.1789 PMC1461583

[B5] Yoshizawa M Gorički Š Soares D Jeffery WR Evolution of a behavioral shift mediated by superficial neuromasts helps cavefish find food in darkness Curr Biol 2010 20 1631 1636 10.1016/j.cub.2010.07.017 20705469 PMC2946428

[B6] Yoshizawa M Ashida G Jeffery WR Parental genetic effects in a cavefish adaptive behavior explain disparity between nuclear and mitochondrial DNA Evolution 2012 66 2975 2982 10.1111/j.1558-5646.2012.01651.x 22946818 PMC3434958

[B7] Rice WR Genetic hitchhiking and the evolution of reduced genetic activity of the Y sex chromosome Genetics 1987 116 161 167 3596229 10.1093/genetics/116.1.161 PMC1203114

[B8] Smith JM Haigh J The hitch-hiking effect of a favourable gene Genet Res 1974 23 23 35 10.1017/S0016672300014634 4407212

[B9] Jernigan RW Culver DC Fong DW The dual role of selection and evolutionary history as reflected in genetic correlations Evolution 1994 48 587 596 10.2307/2410471 28568263

[B10] Culver DC Cave Life, Evolution and Ecology 1982 Cambridge, MA: Harvard University Press 189

[B11] Culver DC Pipan T The Biology of Caves and Other Subterranean Habitats 2009 Oxford, UK: Oxford University Press 254

[B12] Orr HA Testing natural selection vs. genetic drift in phenotypic evolution using quantitative trait locus data Genetics 1998 149 2099 2104 9691061 10.1093/genetics/149.4.2099 PMC1460271

[B13] Yoshizawa M Jeffery WR Evolutionary tuning of an adaptive behavior requires enhancement of the neuromast sensory system Commun Integr Biol 2011 4 89 91 21509190 10.4161/cib.4.1.14118 PMC3073282

[B14] Yamamoto Y Stock DW Jeffery WR Hedgehog signalling controls eye degeneration in blind cavefish Nature 2004 431 844 847 10.1038/nature02864 15483612

[B15] Wilkens H Evolution and genetics of epigean and cave *Astyanax-fasciatus* (Characidae, Pisces) - support for the neutral mutation theory Evol Biol 1988 23 271 367

[B16] Strecker U Hausdorf B Wilkens H Parallel speciation in *Astyanax* cave fish (Teleostei) in Northern Mexico Mol Phylogenet Evol 2012 62 62 70 10.1016/j.ympev.2011.09.005 21963344

[B17] Borowsky R Restoring sight in blind cavefish Curr Biol 2008 18 R23 R24 10.1016/j.cub.2007.11.023 18177707

[B18] Bradic M Beerli P García-de León FJ Esquivel-Bobadilla S Borowsky RL Gene flow and population structure in the Mexican blind cavefish complex (*Astyanax mexicanus*) BMC Evol Biol 2012 12 9 10.1186/1471-2148-12-9 22269119 PMC3282648

[B19] Gross JB The complex origin of *Astyanax* cavefish BMC Evol Biol 2012 12 105 10.1186/1471-2148-12-105 22747496 PMC3464594

[B20] Ornelas-García CP Domínguez-Domínguez O Doadrio I Evolutionary history of the fish genus *Astyanax* Baird & Girard (1854) (Actinopterygii, Characidae) in Mesoamerica reveals multiple morphological homoplasies BMC Evol Biol 2008 8 340 10.1186/1471-2148-8-340 19102731 PMC2657800

[B21] Wilkens H Strecker U Convergent evolution of the cavefish *Astyanax* (Characidae, Teleostei): genetic evidence from reduced eye-size and pigmentation Biol J Linn Soc Lond 2003 80 545 554 10.1111/j.1095-8312.2003.00230.x

